# Supplementary figures and images for: Binary semantic segmentation for detection of prostate adenocarcinoma using an ensemble with attention and residual U-Net architectures
Source: PeerJ Comput Sci. 2023 Dec 20;9:e1767. doi: 10.7717/peerj-cs.1767 (PMC10773872; doi:10.7717/peerj-cs.1767)

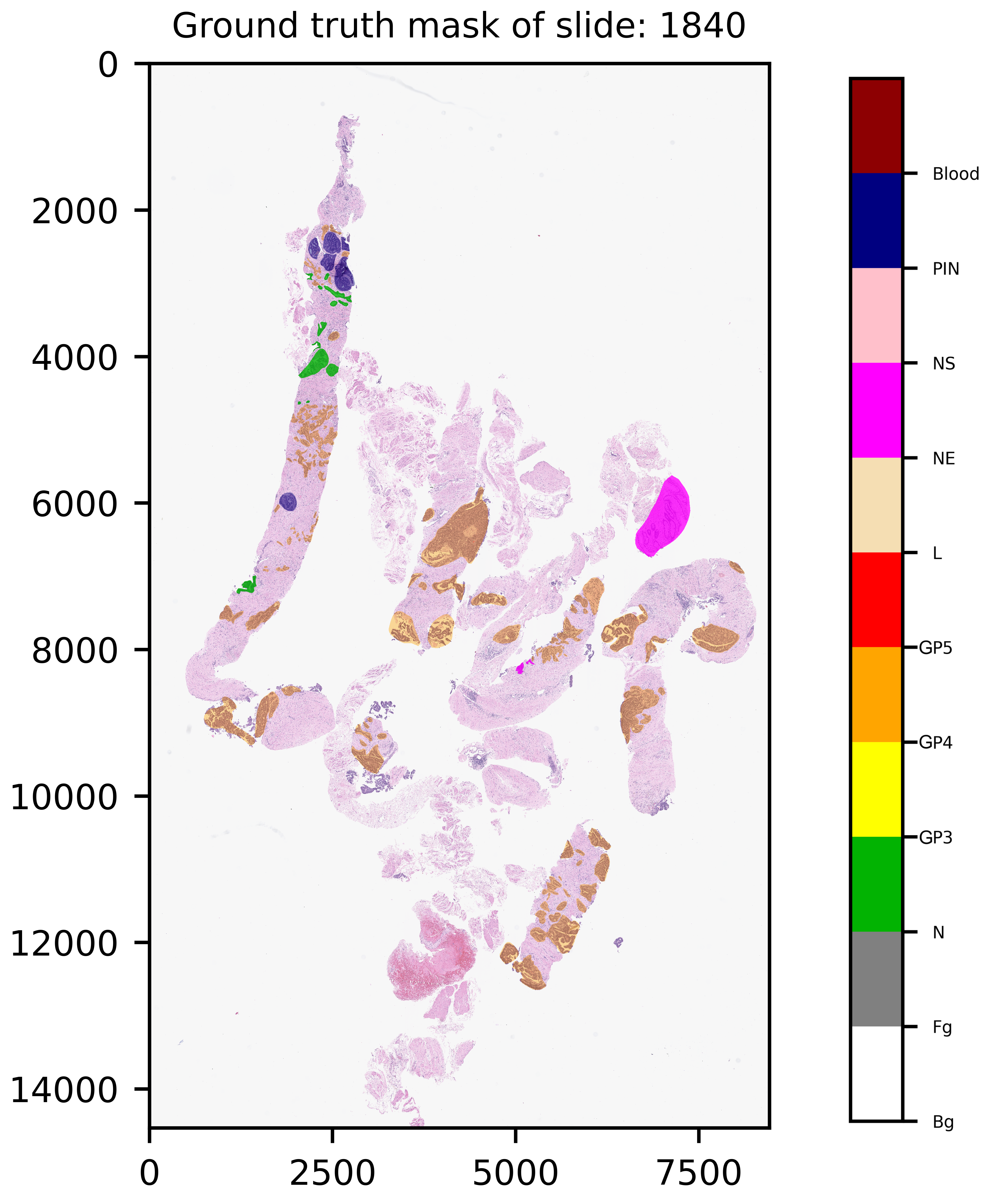

Supplement: Supplemental Information 1 [file peerj-cs-09-1767-s001.png]

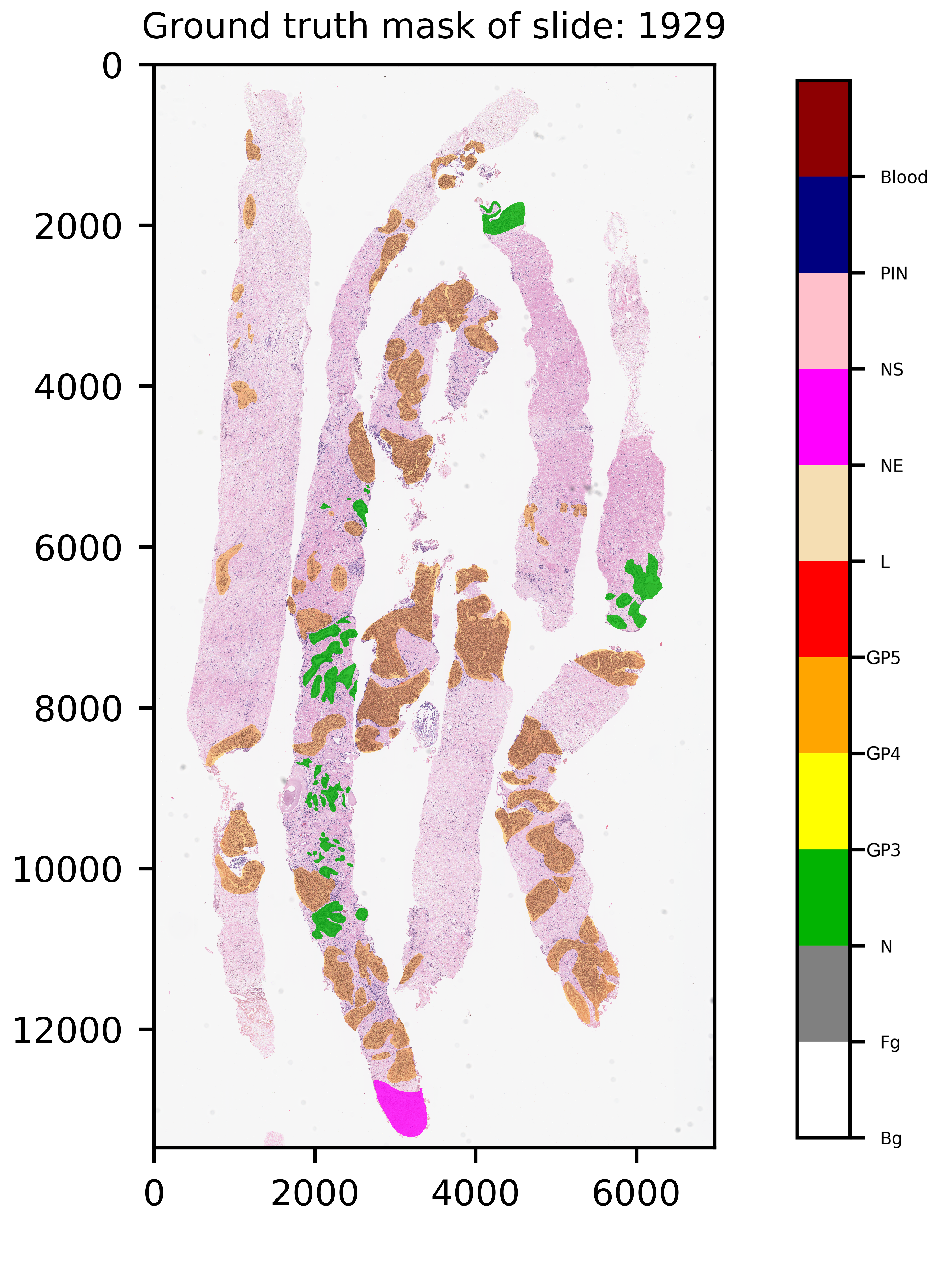

Supplement: Supplemental Information 2 [file peerj-cs-09-1767-s002.png]

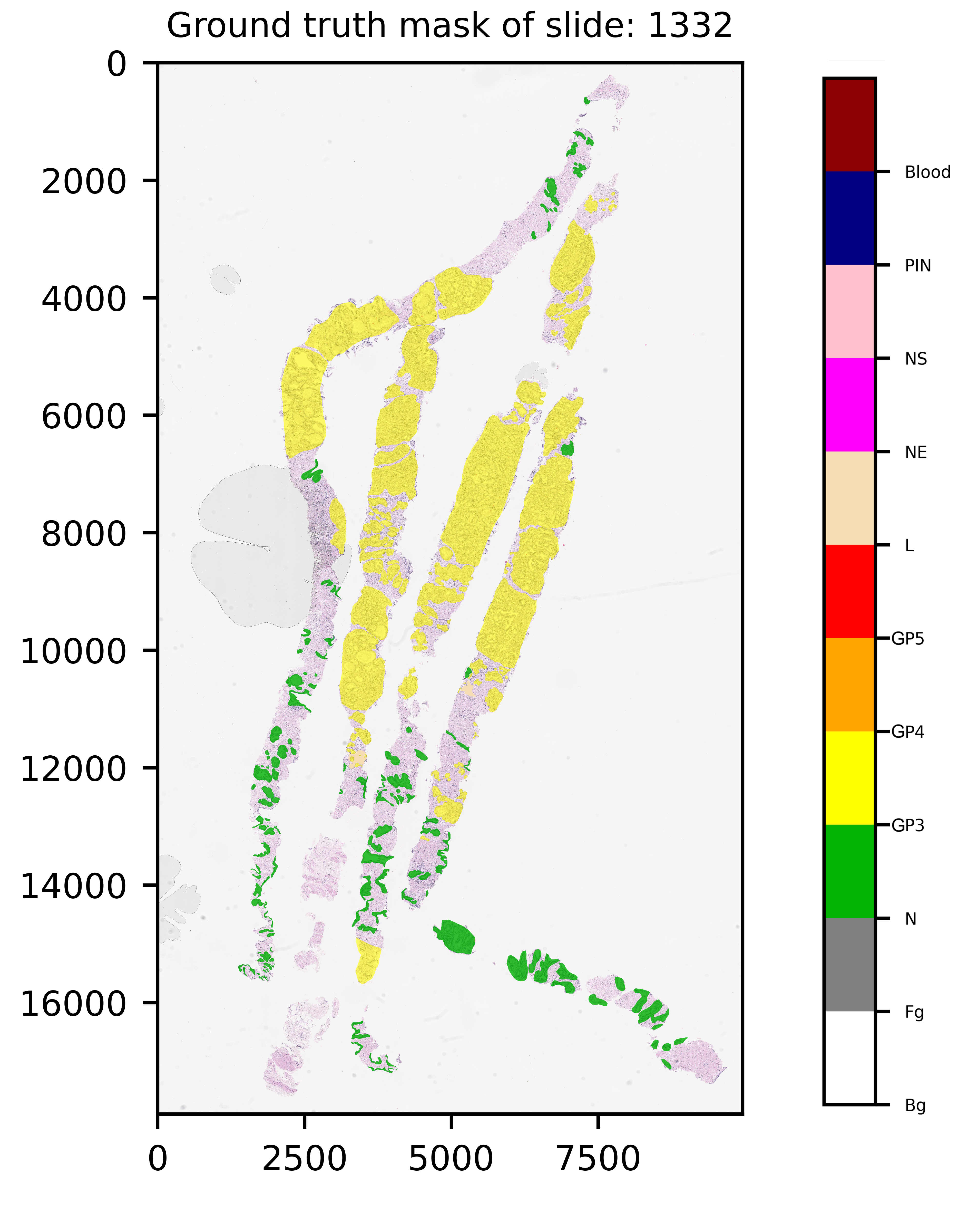

Supplement: Supplemental Information 3 [file peerj-cs-09-1767-s003.png]
