# Supplementary material for: Binary semantic segmentation for detection of prostate adenocarcinoma using an ensemble with attention and residual U-Net architectures
Source: PeerJ Comput Sci. 2023 Dec 20;9:e1767. doi: 10.7717/peerj-cs.1767 (PMC10773872; doi:10.7717/peerj-cs.1767)
Supplement: Supplemental Information 4 [file peerj-cs-09-1767-s004.docx]

Supplementary Materials

Table S1. Hyper-parameters and their configurations of all models for the proposed segmentation approach

| **Parameter** | **Configuration** |
| --- | --- |
| Patch size | 256 x 256 |
| Rescale | 1/255 |
| Kernel size | 3 x 3 |
| Batch size | 16 |
| Epochs | 100 |
| Dropout rate | 0.25 |
| Activation function | ReLu for all CNN-based layers and Softmax for the prediction layer |
| Optimizer | Adam with initialized learning rate |
| Learning rate | 1e-4 |
| Class weights | Utilized |
| Data augmentation | Horizontal flip (selection probability of 0.5) |
|  | Vertical flip (selection probability of 0.5) |
| ReduceLROnPlateau | Monitor on validation of Jacard coefficient with factor = 0.8, patience = 5, min_lr = 1e-4/100 |
| EarlyStopping | Monitor on loss with patience = 9, min_delta = 1e-4/100 |

Table S2 Model parameters and training times

| **Model** | **Parameters** | **Training times** |
| --- | --- | --- |
| U-Net | 31,402,570 | 1 day, 0:49:18 hrs. |
| AU-Net | 37,334,734 | 1 day, 9:22:31 hrs. |
| ARU-Net | 39,090,446 | 1 day, 14:59:32 hrs. |
